# The predictors of spiritual dryness among Iranian cancer patients during the COVID-19 pandemic

**DOI:** 10.3389/fpsyg.2022.1024009

**Published:** 2023-01-20

**Authors:** Azam Shirinabadi Farahani, Sara Hamideh Kerdar, Hadis Ashrafizadeh, Arndt Büssing, Nasrin Mehrnoush, Mohammad Esmaeil Akbari, Maryam Karami, Salehe Tajalli, Leila Khanali Mojen, Maryam Rassouli

**Affiliations:** ^1^Department of Pediatric and Neonatal Intensive Care Nursing, School of Nursing and Midwifery, Shahid Beheshti University of Medical Sciences, Tehran, Iran; ^2^Chair of Medical Theory, Integrative and Complementary Medicine, Faculty of Health; Witten/Herdecke University, Witten, North Rhine-Westphalia, Germany; ^3^Student Research Committee, Faculty of Nursing, Dezful University of Medical Sciences, Dezful, Iran; ^4^Professorship Quality of Life, Spirituality and Coping, Department of Health, Witten/Herdecke University, Herdecke, North Rhine-Westphalia, Germany; ^5^Department of Pediatric Nursing, School of Nursing and Midwifery, Ardabil University of Medical Sciences, Ardabil, Iran; ^6^Cancer Research Center, Shahid Beheshti University of Medical Sciences, Tehran, Iran; ^7^Department of Medical-Surgical Nursing, School of Nursing and Midwifery, Shahid Beheshti University of Medical Sciences, Tehran, Iran; ^8^Nursing Care Research Center, School of Nursing and Midwifery, Iran University of Medical Sciences, Tehran, Iran; ^9^Pediatric Congenital Hematologic Disorders Research Center, Research Institute for Children’s Health, Shahid Beheshti University of Medical Sciences, Tehran, Iran

**Keywords:** spiritual dryness, spirituality, cancer, COVID-19, pandemic, palliative care, Iran

## Abstract

**Background:**

Spiritual struggles affect the wellbeing of religious people. Among them are strugglers with God which is perceived as non-responsive and distant. These perceptions were so far analyzed predominantly in Western societies with a Christian background, but not in Muslims from Iran. The aim of this study was to determine the predictors of spiritual dryness among cancer patients in Iran during the COVID-19 pandemic.

**Methods:**

Cross-sectional study with standardized questionnaires (i.e., *Spiritual Dryness Scale, WHO-5, BMLSS-10, Awe/Gratitude Scale*) among 490 cancer patients (mean age 49.50 ± 14.92 years) referring to the selected educational hospitals in Tehran (the capital of Iran), who were selected through convenience sampling and based on the inclusion criteria, enrolled between December 2020–May 2021. Data analysis was done using SPSS software version 26 and the statistical methods including calculating the mean and the standard deviation, correlation coefficients, as well as regression analysis.

**Results:**

The overall experience of spiritual dryness was perceived regularly in 10.2% of Iranian cancer patients, sometimes in 22.9%, rarely in 22.9%, and never in 43.3%. The mean ± SD was 25.66 ± 5.04, and the scores ranged from 10 to 55. A higher score means greater spiritual dryness. The strongest predictors of spiritual dryness were praying activities Furthermore, the perception of burden due to the pandemic was positively correlated with spiritual dryness. Moreover, each 1 unit increase in its score changed the spiritual dryness score by 0.2 units. The regression of spirituality-related indicators, demographic-clinical variables, and health-related behaviors accounted for 21, 6, and 4% of the total SDS variance, respectively. These findings show that with an increase in praying, performing daily prayers, and the indicators related to spirituality, spiritual dryness will decrease. Most patients were able to cope with these phases often or even regularly, while 31.1% were never or rarely only able to cope.

**Conclusion:**

The results of this study showed that in times of crisis, cancer patients’ faith and confidence in God could be challenged. It is not the disease itself which seems to be associated with this form of crisis, but their religious practices. Therefore, it is necessary to support these patients during their struggle, especially as spirituality is one of the best approaches to cope with the disease.

## 1. Introduction

The most important crisis of the 21st century, the COVID-19 pandemic, has affected societies in various ways (Büntzel et al., 2020; Büssing et al., 2020a,b; Heidari et al., 2020; Büssing et al., 2021a,b). Since COVID-19 is considered a new disease and many things surrounding it are still unknown, it has caused confusion, anxiety, and fear among the general public and has affected the behavior of individuals in societies (Roy et al., 2020). Cancer patients appear to be at a higher risk regarding the above-mentioned emotional reactions and disorders (Hatamipour et al., 2015; Aliyari, 2020) compared to the non-patient population. Therefore, although being anxious is normal for people during and after the pandemic, some cancer patients may experience this challenge stronger than others (Aliyari, 2020). Uncertainty about the future and the disease outcome is common among people with cancer (Sajjadi et al., 2016), which is exacerbated by concerns about COVID-19 and the possible inability to continue the recommended treatments (World Health Organization, 2020). However, these reactions are not specific for cancer patients as these fears were experienced similarly also by non-diseases people (Büntzel et al., 2020; Büssing et al., 2020a,b).

Evidence shows that the spiritual needs of cancer patients increase dramatically (Kamian, 2014). During their experience of life with cancer, when patients are searching for the purpose and the meaning of life and their hope is threatened, the role of spiritual health becomes evident as a vital aspect of having a healthy life (Weber and Pargament, 2014). People’s attention to spirituality as a resource may increase particularly in times of crisis (Rahnama et al., 2012). Cancer diagnosis jeopardizes a patient’s self-confidence and faith and disrupts personal communication due to uncertainty about the future. The previous adjustment mechanisms seem to be inadequate and the individual faces spiritual crisis. When spiritual health is seriously threatened, a person may lose the meaning in life or suffer loneliness or mental disorders such as depression (Kamian, 2014).

Several religious people experience difficulties to rely on their faith or spirituality as a resource. The reasons are complex. Exline et al. (2014) differentiated six domains of spiritual struggles that may affect a person’s mental health: divine (in terms of relation to God), demonic (as causes of negative life events), interpersonal (conflicts with religious peoples and institution), moral (difficulties to follow the moral principles), doubt (in terms of own beliefs), and ultimate meaning (not perceiving meaning in life). Such spiritual crises and conflicts are associated with the feelings of disharmony, disconnection, and the loss of meaning and purpose in life, and can lead to passive reactions such as despair, compulsion, depression, and ultimately indifference (Krakauer et al., 2021). On the other hand, one’s faith can be used as a source of adjustment which leads to spiritual integrity or awakening and coherence (Nemati et al., 2017). Such spiritual crises are at the crossways to either overcoming these struggles or losing faith and thus an important resource to cope (Büssing, 2019). One of the most important adjustment responses among cancer patients is their return to spirituality which plays a significant role in enhancing the adaptation and their quality of life (Kamian, 2014). This interest in spirituality as a resource because of the disease is also found in rather secular societies (Büssing et al., 2009). Improving spiritual health is assumed to be a vital factor in reducing cancer-related tensions, helping them to better spend the rest of their lives with the disease, when patients have access to this resource (Sajjadi et al., 2016).

At the start of the COVID-19 pandemic, scholars assumed that one way to cope with the crises is strengthening faith and relying on this resource (Asadzandi, 2020; Kao et al., 2020; Koenig, 2020; Kowalczyk et al., 2020; Pirutinsky et al., 2020; Barmania and Reiss, 2021; Büssing, 2021). For religious people, making a stronger connection with God can reduce distress, anxiety, and stress and may increase peace and hope, which in turn can enhance immunity against the disease. Therefore, one way to maintain and improve health among different groups of people, including cancer patients, is to benefit from the own spirituality while facing the uncertainty caused by the pandemic (Heidari et al., 2020). Depending on the personality structure and cultural and religious differences, one may or may not use his/her faith to cope with the outcomes of the COVID-19 pandemic the pandemic-related stressors (incl. Feeling lonely and socially isolated) and its burden may influence whether and how one is able to utilize the resource spirituality (Büssing et al., 2022). There are findings that the long phases of lockdown and social distancing have also challenged individuals’ faith and trust in God, with an associated decrease of praying activity and increased proportion of people who stated they have lost their faith during the pandemic (Büssing et al., 2022).

Thus, in times of crisis also religious people may also experience spiritual challenges or even struggles. They may feel that God is distant and does not answer their prayers, or they may feel abandoned by Him, which leads to spiritual ‘*dryness’* and spiritual ‘*emptiness’* (Büssing et al., 2013, 2016, 2017). The notion that prayers go unanswered is usually related to the perceived distance from God (Büssing et al., 2020a,b). The feelings of spiritual emptiness and inability to communicate with God are also an aspect associated with spiritual dryness (Büssing et al., 2013).

Some factors seem to be influential in causing spiritual dryness. Research has shown that less adherence to faith in life, lower sense of coherence, depressive symptoms, and emotional exhaustion are the predictors of spiritual dryness (Büssing et al., 2021a,b). In Seventh-day Adventists, spiritual dryness was predicted by spiritual exhaustion and low perception of the sacred in their life, and further by difficulties in their prayer life, low wellbeing, and emotional exhaustion. As these studies were from people with a Christian background, it was interesting to see whether such phases were also observed in Muslims. Indeed, in a study conducted on Iranian Muslims during the pandemic, spiritual dryness was perceived, too (Büssing et al., 2021c). Here, the use of mood-enhancing drugs (probably to buffer the outcomes of the pandemic-related stressors), social isolation/loneliness because of the lockdowns, and prayer activity (to cope with the stressors) were identified as positive predictors, and restrictions of daily life activities, as a negative predictor of spiritual dryness (Büssing, 2021).

Spiritual struggles, doubts about God and His proximity, as well as the feeling that God does not care about human concerns, are profound human experiences dating back to old times (Büssing, 2019). Therefore, this specific form of spiritual struggle may also be experienced during the COVID-19 pandemic with its phases of social isolation. Cancer patients may be a critical group at risk, as they struggle with the implications of their life-threatening disease, their fears, and worries about their future, their social relations etc., and now with the social outcomes of the lockdowns and fears of own infection with the coronavirus.

### 1.1. Aims and objectives of the present study

Therefore, the aim of this study was to evaluate the predictors of spiritual dryness as a specific form of spiritual crisis among cancer patients during COVID-19 pandemic in order to provide effective care for the patients who experience these phases of specific spiritual struggle. The knowledge who may experience such phases of spiritual dryness and how these patients should be supported is a crucial point, as the topic of spiritual struggles is rather addressed in palliative medicine than in the early phases of the disease where spiritual needs remain often unmet (Balboni et al., 2011; Büssing et al., 2013).

## 2. Methods

### 2.1. Study design

This research is a cross-sectional descriptive correlational study, which was done in the educational hospitals of Tehran (the capital of Iran). Since these hospitals are considered as the referral centers for cancer patients from all over the country, the samples are considered to be almost the representative of the cancer patients in the country.

### 2.2. Recruitment of participants

In this research, the samples were the cancer patients visiting the selected teaching hospitals in Tehran, selected through convenience sampling method, and based on the inclusion criteria.

The inclusion criteria consisted of having cancer, being over 18, definitive diagnosis of cancer (regardless of the type of cancer), being aware of the disease course and treatment, being able to read and write Farsi, and not having mental disorders as stated by the patient. The sample size was calculated using Cochran’s sample size determination formula as seen below:


$$n=\frac{Z_{1-\alpha /2}^{2}\times p\times \left(1-p\right)}{d^{2}}$$

Where, *Z* = 1.96, *p* = 0.5 and *d* = 0.05. Therefore, considering a 10% dropout rate, the minimum sample size was calculated to be 350. Designated 490 subjects completed the questionnaire.

### 2.3. Data collection

In order to conduct this study, the researchers first uploaded the Farsi version of the tool on the Porsline.^1^ Then, the research team sent the research objectives, the informed consent form, and the questionnaire’s link to the patients through WhatsApp, and invited them to participate in the study. The phone numbers of these people were recorded in the patient files, so after expressing their consent, the link of the questionnaire was sent to them.

Data collection was done from December 2020 to May 2021 during the COVID-19 pandemic.

The following questionnaires in Farsi language were used:

Pandemic-related “*burden*” (pressure/stress, anxiety/insecurity, loneliness/social isolation, and the restrictions due to the financial and economic situation caused by COVID-19 pandemic) was measured with 5 numeric rating scales, using a 10-point Likert scale, *not at all* (0–20%), *relatively strong (30–70%),* and *extremely strong* (80–100%), which were combined to one factor (5NRS) as described (Büssing et al., 2020b).

*Wellbeing* was measured using the WHO-5 Well-being Index (Bech et al., 2003). Accordingly, wellbeing was measured in patients based on their condition during the last 2 weeks, being scored on a 6-point Likert scale from *at all of the time* (1) to *no time* (6). The initial score of WHO-5 Well-being Index falls between 0 and 25, which is multiplied by 4 to calculate the final score, where 0 indicates the lowest level of wellbeing and 100, the highest possible level (World Health Organization, 1998; Bech et al., 2003; Topp et al., 2015). The score range is 6 to 30, and a higher score means a lower wellbeing index.

*Life satisfaction* was evaluated using Brief Multidimensional Life Satisfaction Scale (BMLSS) (Büssing et al., 2009). This 14-item questionnaire address five main dimensions of life satisfaction: intrinsic (oneself, life in general), social (friendships, family life), external (work situation, habitation), prospective (financial situation, future prospects), and health situation, abilities to deal with daily life concerns. The scale was scored on a 7-point Likert scale ranging from complete dissatisfaction to complete satisfaction. The psychometric evaluation of this scale in Iran was done by Shirinabadi Farahani et al. (2021). The score range is 14 to 98, and a higher score means greater life satisfaction.

*Awe/Gratitude* as an experiential aspect of spirituality that refers to the frequency of situations where participants experience times of pausing for wondering awe in specific situations with subsequent feelings of gratitude. The 7-item Awe/Gratitude scale (GrAw-7) has good psychometric properties (Cronbach’s alpha = 0.82) and is not contaminated with specific religious or spiritual terminology (Büssing et al., 2018). The items of this scale are scored on a 4-point Likert scale ranging from *never* (0) to *regularly/very often* and transferred to a 100% level. The score range is 0–28, and a higher score means greater awe/gratitude.

*Spiritual dryness* was assessed using the 7-item spiritual dryness scale (SDS-7) which has good internal consistency (Cronbach’s *α* = 0.87) (Büssing et al., 2013). In the Iranian version, the internal consistency was good, too (Cronbach’s *α* =0.79) (Büssing, 2021); three questions were added to the psychometrics of the Persian version. The instrument addresses feelings that God is distant (regardless of the own efforts to draw close to Him), that one’s prayers go unanswered, feelings of being abandoned by God, and finally to be spiritually ‘empty’ or not being able to give any more (both in terms of a spiritual exhaustion). The items were scored on a 5-point Likert scale including *not at all* (1), *rarely* (2), *occasionally* (3), *fairly often* (4), and *regularly* (5). SDS scores are obtained after calculating the mean scores of the scale. The score range is 10–50, and a higher score means greater spiritual dryness.

### 2.4. Translation of instruments

In the present study, other than BMLSS, the other tools were translated and validated based on the approach proposed by Wild et al. (2005). After obtaining written permission from the original developers, the tools were translated into Farsi by two translators fluent in English and Farsi (forward translation). Then, the two translations were compared and after making minor changes in the vocabulary, the final version was prepared. In the next step, the final translated version was given to two other translators who had no contact with the previous ones and were unaware of the process (back-translation). Then, the research team, who were completely familiar with the concepts mentioned in the tools, compared the two back-translation versions of each tool to ensure their similarities with the original versions, and some grammatical corrections were applied in the Farsi version (back-translation review). Next, the psychometric evaluation of the translated tools was done by assessing the qualitative content validity and face validity. To examine the face validity, the research tools were distributed among 15 cancer patients hospitalized in the oncology wards of the selected hospitals across the country with the aim of determining the understandability and the answerability of each item. The content validity was assessed using a qualitative approach. To this end, the tools were provided to 10 experts in the field of nursing, psychology, spirituality, and psychometric, with the aim of evaluating the clarity and the simplicity of each of the items of the questionnaires.

### 2.5. Statistical analysis

SPSS V26 (Armonk, NY: IBM Corp) was used to analyze the data. The normality of the data was examined using Kolmogorov–Smirnov test, with *p* < 0.05 as the statistical level of significance. Descriptive statistics and the indicators of dispersion were used for reporting the frequency and the percentage of variables. Spearman and Pearson correlation tests were used to examine the relationship between demographic variables, *burden*, *wellbeing*, *life satisfaction*, *awe/gratitude,* and *spiritual dryness*. In case of discovering significant correlations, linear regression was used to examine its direction and intensity. Regarding the cut-offs for the correlation coefficients, most researchers would probably agree that a coefficient of <0.1 indicates a negligible relationship, and a coefficient of >0.9 shows a very strong one; values in-between are disputable. In this research, it is based on the same method.

## 3. Results

In this study, 490 cancer patients participated. Their mean age was 49.50 ± 14.92 years, with 18 and 90 as the lowest and highest values, respectively. The demographic and clinical characteristics of the patients are displayed in Table 1.

**Table 1:** Demographic and clinical characteristics of the participants (n = 490).

Variables		*N*	**%**
*Sociodemographic data*			
Age (years)	18–30	58	11.8
	31–40	89	18.2
	41–50	97	19.8
	51–60	122	24.9
	61<	124	25.3
Gender	Female	269	54.9
	Male	221	45.1
Marital status	Single	82	16.7
	Married	350	71.4
	Divorced	22	4.5
	Other	35	7.1
*Clinical data*			
Duration of the disease (year)	Less of 1	237	47.1
	1–3	155	31.6
	3–5	74	15.1
	More than 5	28	5.7
Type of cancer	Gastric and colorectal	111	22.7
	Breast	59	12.0
	Lung	34	6.9
	Blood	107	21.8
	Lymphoma	59	12.0
	Bladder	8	1.6
	Testicles	21	4.3
	Other	91	18.6
Use morphine or other drugs to control pain?	Yes	151	30.8
	No	339	69.2
*Indicators of spirituality*			
Faith as a stronghold	I do not agree	46	9.4
	No idea	137	28.0
	In most cases	307	62.7
Praying	Never	52	10.6
	At least once a month	80	16.3
	At least once a week	90	18.4
	At least once a day	268	54.7
I pray for myself and others in private	Never	15	3.1
	Rarely	87	17.8
	Mostly	169	34.5
	Continuous	219	44.7
I perform the obligatory prayers	Never	49	10.0
	Rarely	80	16.3
	Mostly	109	22.2
	Continuous	252	51.4
*Health behaviors*			
Use of mood-enhancing drugs	Never	402	82.0
	At least once a month	32	6.5
	At least once a week	23	4.7
	At least once a day	33	6.7
Consumption of plant materials (such as Borage, Ginger, …)	Never	158	32.2
	At least once a month	180	36.7
	At least once a week	95	19.4
	At least once a day	57	11.6

Spiritual dryness was perceived regularly by 10.2%, sometimes by 22.9%, and rarely by 22.9% of the participants, while 43.3% of the respondents did not experience it at all. The respective mean SDS score among cancer patients was 25.66 ± 5.04. The frequency and the percentage of the data on spiritual dryness are shown in Table 2.

**Table 2:** Frequency of spiritual dryness perceptions.

Spiritual dryness	Not at all		Rarely		Occasionally		Fairly often		Regularly	
	*N*	%	*N*	%	*N*	%	*N*	%	*N*	%
I have the feeling that God is distant from me, regardless of my efforts to draw close to him	203	41.4	85	17.3	125	25.5	44	9.0	33	6.7
I have the feeling that God has abandoned me completely	281	57.3	80	16.3	76	15.5	47	9.6	6	1.2
I experience times of `spiritual dryness´	212	43.3	112	22.9	112	22.9	44	9.0	6	1.2
I have the feeling that I am “spiritually empty.”	221	45.1	100	20.4	130	26.5	33	6.7	6	1.2
I have the feeling that my prayers go unanswered.	162	33.1	112	22.9	149	30.4	41	8.4	26	5.3
I know the feeling of not being able to give any more.	134	27.3	116	23.7	129	26.3	86	17.6	25	5.1
Additional items:										
I feel a deep yearning for God in me.	13	2.7	44	9.0	104	21.2	133	27.1	195	39.8
I have found ways to deal with these feelings	40	8.2	112	22.9	133	27.1	104	21.2	98	20.0
These feelings inspire me all the more to help others.	47	9.6	87	17.8	139	28.4	133	27.1	84	17.1
After these phases of “spiritual dryness” or “abandonment by God,” I experience a greater spiritual serenity and depth.	53	10.8	40	8.2	123	25.1	131	26.7	39	21.2

Furthermore, in this study, the mean scores of *perceived burdens* related to the pandemic, emotional *wellbeing*, *life satisfaction,* and *awe/gratitude* were calculated to be 8.81 ± 2.85, 16.81 ± 7.16, 64.25 ± 14.25, and 20.47 ± 3.78, respectively.

Regression analyzes were used to investigate the predictors of spiritual dryness (as a depending variable), i.e., demographic and clinical data age, gender, marital status, duration of the disease, type of cancer, use of morphine or other drugs to control pain, and further indicators of spirituality (Faith as a stronghold, praying for oneself and others in private, performing the obligatory prayers) and health behaviors (use of mood-enhancing drugs, consumption of plant materials). However, several of these putative influences were not significantly related to spiritual dryness (data not shown), and were thus not included in the regression model (Table 3). Finally, *Perception of Burden* (due to the pandemic) was the sole significant predictor of spiritual dryness experiences during the pandemic, while wellbeing, life satisfaction, and Awe/Gratitude had no significant influence in this regression model (Table 3). Although spiritual dyness and life satisfaction were inversely correlated (data not shown), this influence was not verified as significant in the regression model, as this variance was absorbed by *Perception of Burden*.

**Table 3:** Results of linear regression analysis between spiritual dryness scale as dependent variable and putatively influencing variables (inclusion approach).

Model	*B*	SE	Standardized beta	*T*	*P*
Constant	26.66	2.09	–	12.75	<0.0001
Perception of burden	0.346	0.093	0.203	3.73	<0.0001
Wellbeing	−0.046	0.039	−0.066	−1.16	0.247
Life satisfaction	−0.030	0.019	−0.090	−1.57	0.117
Awe/gratitude	−0.042	0.067	−0.035	−0.62	0.534
*R* = 0.227 *R*^2^ = 0.051 Adjusted *R*^2^ = 0.041 *F* = 5.08 *p* = 0.001					

Dependent variable: Total score of spiritual dryness scale.

Table 4 displays the results of univariate regression analysis that was conducted to examine the correlation of spiritual dryness with clinical and demographic variables and the indicators of spirituality and health-related behaviors. According to the regression coefficients, the mean score of spiritual dryness is significantly correlated with (a) the demographic variables: being in the age group of <60 years, being married (or other), while duration of disease of type of cancer had no significant influence, (b) spirituality-related indicators: praying at least once a month or at a week, and performing the obligatory prayers, but not private praying, and (c) health-related behaviors: and using herbal medicine at least once a month using, but not uptake of mood-enhancing drugs at least once a month. Given that the level of significance for these variables is below 0.05, it can be said that these variables are the predictors of dependent variables, or in other words, the mean score of spiritual dryness. In this model, the regression of spirituality indicators, demographic-clinical variables, and health-related behaviors account for 21, 6, and 4% of the total variance of the score of spiritual dryness, respectively.

**Table 4:** Predictors of spiritual dryness analyzed in univariate regression.

Outcomes	Total score of spiritual dryness scale				
Parameter	Standardized beta	SE	95% CI for beta	*t*	*p*
*Demographic and clinical data*					
*Age*					
18–30	Ref	–	–	–	–
31–40	−1.571	0.86	[−3.27, 0.12]	**3.28**	**0.036**
41–50	−1.967	0.86	[−3.65, 0.27]	**5.19**	**0.003**
51–60	−2.506	0.83	[−4.14, −0.86]	**9.00**	**0.023**
>61	−1.686	0.80	[−3.26, −0.10]	4.38	0.070
Gender					
Female	Ref	–	–	–	–
Male	0.095	0.48	[−0.86, 1.05]	0.038	0.845
*Marital status*					
Single	Ref	–	–	–	–
Married	−1.93	0.68	[−3.28, −0.58]	**7.87**	**0.003**
Divorced	−0.48	1.32	[−3.08, 2.11]	0.13	0.713
Other	−3.15	1.04	[−5.21, −1.09]	**9.05**	**0.005**
*Duration of the disease (year)*					
Less of 1	Ref	–	–	–	–
1–3	0.306	0.54	[−0.76, 1.37]	0.31	0.982
3–5	−0.227	0.73	[−1.66, 1.21]	0.09	0.757
More than 5	0.026	1.14	[−2.21, 2.26]	0.00	0.576
*Type of cancer*					
Gastric and colorectal	Ref	–	–	–	–
Breast	0.76	0.90	[−1.00, 2.53]	0.71	0.397
Lung	1.81	1.09	[−0.33, 3.96]	2.75	0.097
Blood	0.87	0.71	[−0.53, 2.27]	1.49	0.222
Lymphoma	1.29	0.87	[−0.42, 3.00]	2.17	0.141
Bladder	2.40	1.85	[−1.23, 6.04]	1.67	0.195
Testicles	0.50	1.28	[−2.02, 3.03]	0.15	0.696
Other	0.96	0.75	[−0.52, 2.44]	1.61	0.204
*Use morphine or other drugs to control pain?*					
Yes	Ref	–	–	–	–
No	0.68	0.52	[−0.33, 1.70]	1.72	0.189
*Indicators of spirituality*					
*Faith as a stronghold*					
It is not correct	Ref	–	–	–	–
No idea	−1.64	0.88	[−3.38, 0.09]	3.43	0.059
In most cases	−1.58	0.83	[−3.22, 0.06]	3.56	0.064
*Praying*					
Never	Ref	–	–	–	–
At least once a month	−1.55	0.95	[−3.40, 0.30]	**2.67**	**0.013**
At least once a week	−2.44	0.95	[−4.27, −0.61]	**6.86**	**0.009**
At least once a day	−2.11	0.85	[−3.79, −0.44]	6.13	0.102
*I pray for myself and others in private*					
Never	Ref	–	–	–	–
rarely	−1.70	1.32	[−4.30, 0.90]	1.64	0.436
mostly	−1.62	1.28	[−4.13, 0.88]	1.60	0.205
continuous	−0.99	1.28	[−3.51, 1.51]	0.60	0.200
*I perform the obligatory prayers*					
Never	Ref	–	–	–	–
rarely	−1.80	0.89	[−3.55, −0.05]	**4.08**	**0.006**
mostly	−1.95	0.87	[−3.66, −0.24]	**4.99**	**0.025**
continuous	−2.19	0.80	[−3.75, −0.62]	**7.49**	**0.043**
*Health behavior’s*					
*Use of mood-enhancing drugs*					
Never	Ref	–	–	–	–
At least once a month	1.49	0.95	[−0.38, 3.37]	2.41	0.053
At least once a week	−0.41	1.20	[−2.77, 1.94]	0.11	0.732
At least once a day	−1.85	0.95	[−3.73, 0.02]	3.73	0.120
*Consumption of plant materials*					
Never	Ref	–	–	–	–
At least once a month	0.65	0.56	[−0.44, 1.74]	**1.34**	**0.001**
At least once a week	0.39	0.73	[−1.05, 1.83]	0.28	0.595
At least once a day	2.63	0.81	[−1.03, 4.23]	10.42	0.246

The bold value in table shows the parameters that are statistically significant.

The regression results show that there is an inverse relationship between the age groups of 31–60 years with spiritual dryness; in other words, for one unit of change in the age groups of 31–40, 41–50, and 51–60 years, spiritual dryness will reduce by 1.57, 1.96, and 2.5, respectively. In addition, for one unit of change in marital status, as well as the individuals who have chosen “other” as their marital status, spiritual dryness will reduce by 1.93 and 3.15. Besides, there was an inverse relationship between performing salah and spiritual dryness. In other words, with one unit of change in prayer once a month, once a week, and once a day, spiritual dryness decreases by 1.55, 2.44, and 2.11, respectively. Spiritual dryness decreases by 1.80, 1.95, and 2.19, respectively, as the result of performing daily salah rarely, often, and constantly. To interpret the regression results, it can be said that the married individuals belonging to the age group of 51–60 years who perform salah at least once a week and perform daily salah steadily experience less spiritual dryness compared to others.

Most patients stated that they have found often or even regularly ways to cope with these phases of spiritual dryness (41.2%), 27.1% at least sometimes, while 31.1% were less able (never or rarely only) (Table 2). When these phases were overcome, most stated “greater spiritual serenity and depth” (47.9%) and the intention to help others more the more (44.2%) (Table 2).

## 4. Discussion

This study aims to determine the predictors of spiritual dryness among cancer patients during the COVID-19 pandemic in Iran. According to the findings of the current study, phases of spiritual dryness were often or even regularly perceived by 10.2% of the patients with cancer. This percentage is lower compared to the findings among a more general population of Iran during COVID-19 pandemic where these phases were perceived often to regularly by 27%, occasionally by 35%, rarely by 23%, and not at all by 15% (Büssing, 2021). In addition, the current findings are close to the rate reported by Catholic priests (Büssing et al., 2017), religious brothers and sisters (Büssing, 2019), non-ordained Catholic pastoral workers (Büssing et al., 2016), Seventh-day Adventists from Germany (Büssing et al., 2021a), and Catholic lay persons from Italy (Büssing et al., 2018). So far, the highest SDS scores were observed in patients with depressive and addictive diseases. Thus, apart from cultural and religious differences, phases of spiritual dryness may be experienced by (probably) all religious people.

Many cancer patients rely on religious beliefs as a source of hope and strength due to the ambiguities of the current situation and the uncertain future, and are often better able to cope with the fear and the loneliness caused by the disease (Surbone and Baider, 2010; Agha Hosseini et al., 2011). However, religious life knows more than only the positive aspect, as also dark phases and religious struggles are part of it (Büssing et al., 2013; Exline et al., 2014; Büssing, 2019). The triggers and promoter of these phases are complex and include psychological, social, and intrinsic factors (Büssing et al., 2021d). When these phases are overcome, many people report deeper spiritual depth and serenity and the intention to help others (Büssing et al., 2017, 2021b), as it was found in this Iranian sample, too. This process could be interpreted as spiritual growth or spiritual transformation that is associated with compassion and psychological wellbeing. In fact, spiritual health is a complicated process that often requires of psychosocial and spiritual growth, accompanied by ups and downs while encountering difficulties of life (Mirhosseini et al., 2020). Such experience is also observed among the family caregivers of Iranian cancer patients. The long treatment procedure and not having the expected results of treatment leads to a contradiction and disharmony in the caregiver’s hope, values, and beliefs about God. Consequently, this results in various forms of spiritual crisis such as the inability to explain the reasons of the disease, doubting the divine justice, and seeing the disease as God’s punishment for one’s previous sins and wrongs deeds, which keeps the caregivers away from spiritual and religious rituals and practices, and restricts their connection to God (Nemati et al., 2017; Khademi et al., 2019).

In this study enrolling cancer patients, we have seen that most are able to cope with these phases of spiritual insecurity and dryness, while 31% were less able to cope with it. Particularly this group of patients requires adequate support that takes their experiences serious. The experience of these phases of spiritual dryness is not unexpected and is not necessarily a result of one’s *weak faith* or *failure* (Büssing et al., 2020a,b; Büssing, 2021; Büssing et al., 2021a,b). It is a ‘normal’ process during the religious life. Some theologians regard this experience as essential for spiritual growth. However, what about those who patients have lost their hope and lost their faith in God? - Here, empathetic support from psychotherapists and pastoral worker is necessary.

According to the results of this study, perceived burden related to the pandemic is the best predictor of spiritual dryness among the patients with cancer. The concept of pandemic-related burden refers to perceived limitations in daily life as being under pressure/stress, anxiety/insecurity, loneliness/social isolation, and the limited pandemic-related economic and financial condition. In a study on the general population of Iran, among the stressors associated with COVID-19 the perception of being restricted in the daily life activities had a weak negative relationship with spiritual dryness, while loneliness/social isolation and financial limitation had a weak positive relationship with spiritual dryness (Büssing et al., 2021c). Instead, being stressed and under pressure or the feeling of fear and insecurity had no significant relationship with spiritual dryness (Büssing, 2021). The further results of a study on patients with multiple myeloma during the COVID-19 pandemic showed that these patients have many concerns regarding the future and their family, friends, and communicating with their spouses; however, the upcoming events are very vague (Sweeney and Ahlstrom, 2020). In addition, studies have shown that patients with malignancies experience higher levels of distress, anxiety, and depression than general public. A longer course of treatment is also associated with greater anxiety (Pitman et al., 2018; Tsaras et al., 2018; Slimano et al., 2020), and depression have increased during the pandemic (Momenimovahed et al., 2021). Spiritual dryness also had a moderate negative correlation with the symptoms of depression, anxiety, and the perception of stress (Büssing et al., 2016). Evidence shows that the higher the perceived level of stress, anxiety, and depression, the lower one’s ability to adjust to different situations, and the greater the perception of spiritual dryness (McCoubrie and Davies, 2006; Rezaei et al., 2009; Bastani et al., 2014).

Another predictor of spiritual dryness was the use of spirituality-related activities (performing obligatory prayers, but not private prayers for oneself or others). Therefore, those who performed salah or prayed daily had the lowest scores of spiritual dryness, which reflects their religious confidence and shows that their connection with God is more stable. On the other hand, the participants who prayed or performed salah only once a week or once a month had the highest scores of spiritual dryness, indicating that their connection with God may either not be stable or that they perceive that their prayer remains unanswered (which is an aspect of spiritual dryness) and they are thus pray less often. Difficulties in prayer life are one of the crucial aspects of spiritual dryness, as prayers are assumed not to be answered by God, and may thus be considered as ‘useless’. Difficulties in prayer life were also found in Seventh-day Adventists as relevant predictor (Büssing et al., 2021a). However, patience (or even tenacity) in prayer is nevertheless one of the criteria that the relation with God is still in the forefront, even when one may experience phases of insecurity (Büssing, 2019). Commitment to religion, performing religious practices, and seeking the sympathy of individuals or groups have always been reported as ways to cope for cancer patients (Aquino and Zago, 2007). In a study conducted in Iran and New Zealand, Nejat et al. (2017) showed that cancer patients’ religious sources (e.g., faith in divine destiny or considering the disease as a blessing from God) and performing religious practices were used as strategies to cope with their cancer. These results were similar in both countries, even though the structure of Iran is based on religion and New Zealand is a secular country (Nejat et al., 2017). In this regard, German cancer patients’ interest in spirituality was relatively low during the pandemic, while their perception of nature as a source to face the Sacred was more relevant as a source that relates to psychological wellbeing (Büssing et al., 2020a,b). In the Iranian society, performing salah and praying are considered as the most important strategies to cope with stressful conditions caused by chronic diseases (Livneh and Antonak, 2005; Shirinabadi Farahani et al., 2021), because it leads people’s mind and heart to spirituality and helps them pay attention to the depths of their being and the possibility of ‘healing’ through inner peace and spiritual comfort. Cultural and religious differences may account for these obvious differences. A vital prayer life as observed in Muslim participants may also result in calmness, certainty, belief in the facilitation of things, and self-confidence leading to reduced stress (Khodayarifard and Asayesh, 2020). Muslim cancer patients believe that their recovery and the disease outcome are in the hands of God and that religious activities help them fight cancer (Nejat et al., 2017).

In this study, there was a positive relationship between spiritual dryness and use of herbal medicines at least once a month (also for usage of mood-enhancing medication). The relationship between psychiatric drugs and spirituality is unpredictable and the effects vary from person to person. In the study on the general population of Iran, the use of mood-enhancing drugs and frequency of meditation was considered as a strategy to cope with pandemic-related restrictions, and its correlation with spiritual dryness was reported to be positive (Büssing et al., 2021d). Feelings of social isolation, depressed mood states, and emotional or spiritual stress may call for alternative treatments. In fact, higher levels of perceived stress, anxiety, and depression result in higher tendency to take sedatives and mood-enhancing drugs (Gilan et al., 2015; Margdari Nejad et al., 2017). However, the association with herbal medicine is difficult to explain and may be interpreted as an additional (alternative) approach of hope to fight cancer.

Based on the findings of the current study, a negative correlation was observed between spiritual dryness and life satisfaction; however, this variable was not significant in the regression model. Low life satisfaction was less predictive as compared to perceived burden (“Corona Stressors”), Low satisfaction with life impacts spiritual wellbeing and may affect relationship with God and will thus trigger the perception of spiritual dryness (; Büssing, 2021). In a study from Iran, a strong relationship between life satisfaction of cancer patients and spiritual health was observed (RezaieShahsavarloo et al., 2016). Although the direction of effects is unclear, these findings may imply that patients with better spiritual health can better use their spiritual resources to cope and this, in turn, may have positive effects also on their spiritual health (RezaieShahsavarloo et al., 2016). Distraction from core beliefs of faith may cause some persons to suffer more from inner conflicts and they perceive feeling of hopelessness in the face of crises, and they are thus unable to tolerate deprivation and adversity, and, as a result, the level of life satisfaction will decrease (Sadeghi et al., 2010). However, further studies are needed to confirm the direction of the underlying relationship between life satisfaction and spiritual dryness in cancer patients. Results from this study indicated that both dimensions are not necessarily related. Life satisfaction refers to other dimensions which are not related to the perception of God. The results of the study show that there is a direct and significant relationship between the item SDS0 (I feel a deep yearning for God in me) with the life satisfaction score. That is, the deeper a person feels a connection with God, the more satisfied he is with life. This could mean that in Iranian cancer patients, phases of spiritual dryness would not necessarily affect their general satisfaction with life.

Another factor with an influence on the perception of spiritual dryness was age. The relationship between these two variables was observed to be inverse. In other words, as age increases, spiritual dryness may decrease. This could indicate that with increasing life experiences they have better learned to cope with these phases, while the younger patients might be more disturbed by these experiences as they may assume that God has left them in their fight against the cancer. In this study, the lowest scores of spiritual dryness were obtained among 51 to 60-year-old patients, while no correlation with spiritual dryness was seen in the patients over 61 years. Rezaei et al. (2009) showed that most cancer patients over 70 have a high level of spiritual health (Rezaei et al., 2009), a finding which is also confirmed by the results of other studies (Rowe and Allen, 2004; Omidvari, 2009). In general, it can be said that attraction to spirituality is considered to be a result of aging because it is how one faces the reality of death and adjusts to it (Zimmer et al., 2016).

Finally, a negative correlation was observed between marital status and spiritual dryness. In other words, people who are married experienced more spiritual dryness than single and divorced people. This finding was surprising. In a study on chemotherapy patients in Iran, spiritual health had a higher level among divorced people and those whose spouses had passed away (Rezaei et al., 2009). However, studies show that divorced people and those who are not properly satisfied with their marriages suffer from health problems, and of course, the effect of age and gender should not be ignored (Riley et al., 1998; Karren et al., 2013).

### 4.1. Study limitations

One of the limitations of the current research is the impossibility of making a comparison between pre and post-pandemic circumstances, especially with the aim of determining the predictors of spiritual dryness. Due to the cross-sectional design of the study, not causal interpretations are possible. Due to the pandemic, people with low wellbeing may experience phases of spiritual dryness stronger than those who are emotionally more stable; yet it may also be true that persons who struggle with God because of the disease, experience the negative outcomes of the pandemic stronger than persons who have strong faith in God.

Further, due to the recruitment process not all social groups may have been reached in a similar manner. Therefore, we do not assume that the data are representative for all cancer patients in the Iran.

## 5. Conclusion

The results of this study showed that spiritual dryness is often to regularly experience by 10% of patients with cancer. There is a significant relationship between the mean score of spiritual dryness and age, marital status, prayer activity, the perception of burden, and use of herbal medicine at least once a month. Given that the present study was conducted during the corona pandemic, results showed that in times of crisis, faith and trust of individuals in God might be challenged. Here, the best predictor of spiritual dryness was the perception of corona-related burden, not health and disease-related variables. In this regard, it is necessary to pay attention to the spiritual needs of cancer patients who have experienced an additional challenge during the pandemic alongside their intractable disease, particularly as a large fraction of patients are less able to cope with these faith disturbing experiences. In order to provide a holistic intervention for them and recognize their spiritual dryness while providing comprehensive care. Addressing the psychological and spiritual needs of the patients experiencing spiritual dryness can help to improve their quality of life and wellbeing, especially in situations where any certainty seems uncertain.

## Data availability statement

The raw data supporting the conclusions of this article will be made available by the authors, without undue reservation.

## Ethics statement

The studies involving human participants were reviewed and approved by Research involving human participants have been performed in accordance with the Declaration of Helsinki and code of ethic from Institutional review board approval was obtained from the Ethics Committee of the Cancer Research Center of Shahid Beheshti University of Medical Sciences, Tehran (IR.SBMU.CRC.REC.1400.019). The patients/participants provided their written informed consent to participate in this study.

## Author contributions

AF, HA, SK, AB, NM, MA, MK, ST, LM, and MR designed the study. AF, AB, SK, LM, and MR supervised and directed the study. HA, MK, NM, MA, MK, and ST carried out the implementation. HA and AF processed the experimental data, performed the analysis, and drafted the manuscript. SK, AB, HA, and MR aided in designing the study and worked on the manuscript. All authors discussed the results, commented on the manuscript, and approved the final manuscript.

## Funding

This study was conducted as research project by the vice-chancellor for research affairs of Shahid Beheshti University of Medical Sciences.

## Conflict of interest

The authors declare that the research was conducted in the absence of any commercial or financial relationships that could be construed as a potential conflict of interest.

The reviewer DR declared a past co-authorship with the author AB to the handling editor.

## Publisher’s note

All claims expressed in this article are solely those of the authors and do not necessarily represent those of their affiliated organizations, or those of the publisher, the editors and the reviewers. Any product that may be evaluated in this article, or claim that may be made by its manufacturer, is not guaranteed or endorsed by the publisher.

## Footnotes

^1^https://porsline.ir/an online survey system widely used in Iran in academic studies.
